# Horizontal-vertical illusion in mental imagery: quantitative evidence

**DOI:** 10.3389/fnhum.2015.00033

**Published:** 2015-02-06

**Authors:** Jelena Blanuša, Sunčica Zdravković

**Affiliations:** ^1^Laboratory for Experimental Psychology, Faculty of Philosophy, University of BelgradeBelgrade, Serbia; ^2^Laboratory for Experimental Psychology, Faculty of Philosophy, University of Novi SadNovi Sad, Serbia; ^3^Department of Psychology, Faculty of Philosophy, University of Novi SadNovi Sad, Serbia

**Keywords:** visual imagery, visual illusions, mental image size, gender differences, the horizontal-vertical illusion

## Abstract

The present study had two main goals: (1) to investigate the difference between perception and mental imagery using a visual illusion as a stimulus; (2) to inspect gender related differences in perception and imagery. Our main hypothesis, that there would be no differences between perception and mental imagery, was motivated by previous neuroimaging data. Unlike these neuroimaging studies that demonstrate great similarity between the two processes, results obtained in behavioral studies have not always been consistent. We assumed that this inconsistency was a consequence of methodological differences. Hence, we explored the two processes with a modified behavioral procedure. The additional exploration of gender differences was motivated by the discrepancy between our findings and the existing literature. In two experiments, participants estimated the lines constituting the horizontal-vertical illusion, either in perception or imagery task. Results confirmed that there was no significant difference between perception and imagery: the illusion was equally strong in both tasks. In the second experiment, an additional factor was tested, stimulus size. The results showed that, although there was no significant difference in illusion strength, there was a gender difference in the size of mental image for medium and large stimuli. While male subjects performed equally in the two tasks, female subjects tended to underestimate size in the imagery task. This tendency intensified as the stimulus size increased. Our results not only inform us about the status of illusions in imagery but also offer some answers about the spatial nature of mental representations. We hope that such precise measurements of mental representation might provide better understanding of reasoning that uses mental images.

The attempt to understand mental imagery inspired a long and famous debate. One camp, led by Stephen Kosslyn, described imagery as mental representations analogous to perceived stimuli (Kosslyn, 1973). The other camp, led by Zenon Pylyshyn, theorized that mental images were in fact decomposed into propositions and symbolic in nature (Pylyshyn, 1973). Converging evidence from cognitive neuroscience supported the first proposal, systematically showing that the brain areas used in visual mental imagery overlap with the areas used for visual perception (i.e., cortical areas, such as parietal and frontal; Kosslyn et al., 1997; Ganis et al., 2004). Nevertheless, the results for primary visual areas were not always consistent and sometimes showed differences in activation for perception and imagery (Kosslyn et al., 2001). Taken together, the ratio between studies showing activation vs. no activation in primary visual cortex during imagery are 21:3 for fMRI, 11:13 for PET and 2:7 for SPECT studies. Further studies have shown that not only are these areas active during imagery, but repetitive transcranial magnet stimulation (rTMS) temporarily disabling V1, also disrupts imagery, suggestive of a causal link (Kosslyn et al., 1999).

Hence, overall neuroimaging studies demonstrated that the same brain areas were involved in both tasks. Unfortunately, this involvement might be a consequence of some other property of the task (i.e., spatial layout of the visual mental images) leaving the question of the similarity of underlying mechanisms of the two phenomena still unanswered. Therefore only behavioral tasks can establish similar phenomenology in imagery and perception, confirming the similarity of processes. Experiments using visual illusions as stimuli for imagery and perception proved to be very useful in this respect. For example, demonstrating that one can inspect an imagined “picture” to experience an optical illusion in a similar way as in perception would show the necessary similarity among the processes (Chambers and Reisberg, 1985).

Visual illusions are often just simple geometrical configurations, which generate percepts that do not fully represent physical properties of the stimulus. Typically, illusions are not thought to be influenced by higher visual or cognitive processes (Harris et al., 2011). In fact, illusions are still perceived even when we know all about them. There is a general consensus that illusions occur at a basic, low level of visual processing (for earlier literature see also: Fisher, 1969; Robinson, 1972). It is thus interesting to examine whether illusions also occur in imagery. If illusions can be experienced in imagery, it might suggest that imagery also uses basic low-level vision strategies. If illusions are also of the same magnitude in imagery and perception, this could be a strong indicator that the two processes share more basic mechanisms.

Unfortunately, there were discrepancies in results obtained using illusions as the stimuli in mental imagery tasks. While some reported illusions formed in imagery (Berbaum and Chung, 1981; Wallace, 1984a,b; Ohkuma, 1986), others did not (Reisberg and Morris, 1985; Giusberti et al., 1998). We believe that this discrepancy is both a consequence of different methodologies used in different studies and different criteria for successful creation of illusion in imagery.

One of these studies (Wallace, 1984b), which provided support for the existence of perceptual illusions in imagery and the similarity of perception and imagery processes, like ours investigated the horizontal-vertical illusion. In this study participants were invited to observe the two lines creating the illusion, imagine them, or would be presented with either one of the stimuli lines and be asked to imagine the other. They were instructed to imagine a line of the same size as the presented line, but depending on a condition, sometimes in the same and sometimes in the different orientation. The participants were then asked to provide a magnitude estimate of the illusion. The perception condition, with both stimulus lines presented, showed stable illusory effect for all the participants (1.9 cm difference on the 12 cm targets). The imagery results divided the sample into two groups: (1) the group of gifted imagers who systematically, though non-significantly, amplified the illusion; and (2) the group of non-gifted imagers who could not provide responses in any condition involving either full or partial imagery.

As just mentioned, a typical procedure involved asking participants to imagine two lines of equal length and then inviting them to judge whether they are equal in length.^1^ Already Predebon and Wenderoth (1985) noted that a significant methodological flaw of Wallace’s studies was an ambiguous instruction. It seemed that the lines were “equal and not equal” at the same time (Predebon and Wenderoth, 1985). Moreover, this kind of instruction could be easily biased by the experimenter’s suggestion or expectation, and would seem to be the best candidate for explaining the discrepant results.

A second and related problem was the conception of the imagery task in the early studies. For example, in the just cited studies by Wallace (1984a,b), participants were asked to estimate an illusion that was partly presented (as a picture) and partly imagined. In such a case, the illusion is a hybrid of two very different processes: perception and imagery (or more precisely *memory*, given that imagery is in fact a form of memory task). At the time this hybrid procedure was successfully applied to, for example, the autokinetic (Wallace, 1980), Poggendorff (Goldstein and Weintraub, 1973) and Miller-Lyer illusions (Berbaum and Chung, 1981). The dominant theory at the time was functional equivalence theory (Finke, 1980), highly supportive of the described procedure postulating an easy substitution of an image (formed in imagery) for a physical stimulus. But to put it in Finke’s own words “Effects produced when images are formed are often smaller than corresponding effects produced when objects and events are observed, and vivid imagers often show larger effects when forming images than nonvivid imagers”. This statement precisely characterizes results such as the one previously described (Wallace, 1984b).

In our view, given such differences between perception and imagery, the dual nature of the hybrid procedure is problematic if we want to make really general claims about imagery process vs. perception, claims about the entire population (not just gifted minority) and especially if we want to measure the size of this illusory effect in imagery *per se*. In addition, the following years witnessed very different findings about the role of memory in size representation and perception (Freyd and Finke, 1984; Intraub et al., 1996). These studies demonstrated a more sophisticated role of memory in size estimation (which will be further presented in the Discussion and compared to our results).

Another general methodological issue has been high stimulus complexity. Giusberti et al. (1998) used the Ebbinghaus illusion, constructed of 14 circles in three different sizes and presented partially in three successive frames. Participants had to unite all of these separately presented elements into a single figure in imagery. Firstly, it is not even known whether the mental images could be accurately constructed and combined from previously presented parts. But more importantly, how could performance in perception and imagery be compared in this kind of task?

When it comes to stimulus presentation and control, it should be noted that the possible influence of afterimages on the results was never considered nor controlled (like for example in the domain of color, see Finke and Schmidt, 1977). The main characteristics of experimental procedures include figures first fully shown and then imagined (Giusberti et al., 1998), or figures that are partly viewed and partly imagined (Wallace, 1984a,b; Reisberg and Morris, 1985). Under such presentation conditions, the occurrence of potentially distracting afterimages seems almost inevitable.

In sharp contrast to the usual procedures in visual perception experiments, the above studies used only illusory figures and did not use any control stimuli. Furthermore, those stimuli were not varied in size or any other relevant dimension, there was no manipulation of key parts of the figure, and inevitable clustering of answers in the same direction might well have occurred.

And although we turned to behavioral research to gain a level of understanding that neuroscience seemed unable to provide, measurements used in these studies has failed to deliver a full account of the nature of mental images. That is, only arbitrary and relative measurements were used and the only evidence of an illusory figure’s existence was subjective judgment. Participant were either asked to decide whether the targets were equal or bigger (Wallace, 1984a,b; Reisberg and Morris, 1985) or to make a judgment based on arbitrary units (Giusberti et al., 1998). Therefore, no direct quantitative measurement of imagined targets has ever been obtained.^2^

Many of the results presented above were analyzed in relation to scores on the Vividness of Visual Imagery Questionnaire (VVIQ; Wallace, 1984a,b; Reisberg and Morris, 1985; Giusberti et al., 1998). In those studies results were analyzed and explained separately for high- and low-imagers. Given that we are trying to establish the link between perception and imagery, this conception of scoring can only lead to confusion. Not only is there no analogous example in perception, but also observations from these studies are unlikely to provide a general explanation of imagery process.

We believe that the discrepancy among previous behavioral studies is a consequence of the methodological problems we have outlined. In the present study the methodology was modified and the procedures were simplified, as listed below.

## Simplified, non-ambiguous stimuli

All of our stimuli were non-ambiguous and constructed of simple straight lines. Initially, we started our experiments using three famous illusions: the horizontal-vertical illusion, the Ponzo illusion and Koffka’s illusion. However, analysis of pilot data showed that only the first example was really non-ambiguous and usable in our setup (this was already reported in the literature, see Peterson et al., 1992). Therefore only this illusion will be presented and analyzed in the current paper.

## Simplified imagery task

We used an imagery task that avoided potential problems with the construction of mental images. This task included only visualization of previously presented stimuli. Moreover, the task was constructed in such a way as to avoid the two issues that previous research struggled with. Our stimuli were either viewed or imagined, which prevented construction of hybrid images. This further enabled the use of a simple matching scale, and a simple psychophysical procedure in the experiment.

## Precise measurement on a scale

For the first time in imagery research a matching scale was used, providing not only quantitative measurement and well-defined experimental procedures, but also avoiding previously used paradoxical instructions. Finally, since the scale was used both in perception and imagery it allowed for a simple quantitative comparison between the formed percepts and mental images.

## Prevention of the afterimages

We used a white noise mask to avoid the potential influence of afterimages. The presence of the mask also enabled us to run a large number of independent trials and to collect a large number of measurements for each target in each task.

## Collecting data for both high- and low-imagers

Some of the previous studies used only so-called high-imagers, that is, people gifted for mental imagery. However, in order to expose the actual relation between perception and imagery processes, we cannot restrict the population in only one of the tasks. Our choice of stimuli proved optimal to satisfy this condition, as all participants were able to imagine our stimuli (which we additionally confirmed in the debriefing procedure).

## Wide variety of stimuli

Introducing control sets of stimuli enabled us to control for participants’ answering strategies. Namely, some participants, when presented with a well-known illusion, might pretend that it does not affect them and call the targets equal. However, it is not possible to detect such answering strategy when the targets are really physically equal. Furthermore, it prevents us from distinguishing between this strategy and genuine absence of illusion (which might happen in imagery task).

Consequently, we constructed two control sets of stimuli in order to examine whether all participants followed experimental procedure and to understand why some of the participants did not report experiencing an illusion. Both control sets were constructed in the same manner as illusions, but the crucial geometrical parts involved in the illusory effect, were made physically different sizes. This was done either in the direction of the illusory effect, creating a first control set *pseudo-illusions*, or in the opposite direction, creating a second control set *distractors*. If participants reported “no difference” for control sets, we could exclude them because they failed to follow the procedure.

## Experiment 1

### Method

#### Participants

Twenty-seven undergraduates (14 males and 13 females) from the Faculty of Technical Sciences, University of Novi Sad, participated in the experiment. All had normal or corrected to normal vision and were naïve regarding the experimental hypotheses. They received course credit for their participation and signed a consent form prior to the beginning of the experiment.

#### Stimuli

**Figure 1:**
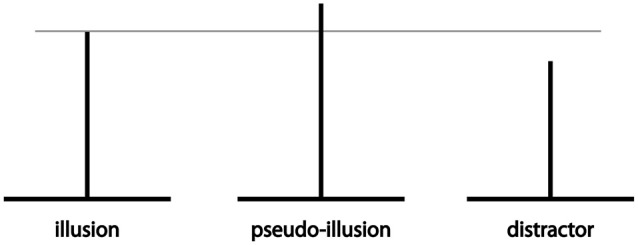
**Types of stimuli used in the study**. Vertical line length was varied in the case of pseudo-illusions and distractor.

Stimulus consisted of two black lines (44 mm long, 1 mm wide) perpendicular to each other to create the horizontal-vertical illusion (Figure 1, left). In the horizontal-vertical illusion those two equal lines appear to have different length due to their orientation (Avery and Day, 1969). Stimuli were presented on a white background (dimensions 112 × 88 mm).

Two control sets of the stimuli, made of physically different lines, were also used. Pseudo-illusions (Figure 1, middle) had the vertical line longer than the horizontal line (see Table 1), so the stimulus was changed in the direction of the illusory effect. As a result, the lines were not only perceptually different—the vertical line was physically longer. If participants still reported the two lines as equal, it would be a strong indicator of answering following a “familiar with the illusion strategy”. Those participants would be excluded from further analysis. Distractors (Figure 1, right) on the other hand had a longer horizontal line (see Table 1). Consequently, the figures did not look like the horizontal-vertical illusion. They were used to avoid systematic answering in the same direction and also to detect inattentive participants.

**Table 1:** **Size of lines for pseudo-illusion and distractor stimuli (in millimeters)**.

Stim. No.	Pseudo-illusions	
	Vertical	Horizontal
1	50	47
2	38	35
3	47	38
4	32	29
5	26	23
	**Distractors**	
1	41	50
2	23	50
3	32	50

*Stimuli size was always 44 mm*.

#### Scale

A scale with vertical lines of 10 different lengths was used for matching. The shortest line was 23 mm, and the longest line was 50 mm, with each line being 3 mm longer than the previous.

#### Procedure

The experiment was conducted in a completely dark room and upon arrival each participant was seated 85 cm from the computer screen. Their task was to match the size of the two lines that produced the stimulus (such as those shown in Figure 1).

SuperLab pro 2.0 for Windows was used for stimulus presentation and recording of responses. Stimuli were presented on a calibrated computer screen (ViewSonic CRT PerfectFlatTM), resolution 1152 × 864 pixels, frequency at 75 Hz). The stimulus background was white, subtending 9.5° × 7.1° degrees of visual angle, while the stimulus lines were 3° long. Participants responded by pressing keys marked 1–10 on a regular computer keyboard.

Each participant was randomly assigned to one of the two experimental tasks: perception or imagery. For the two different tasks we constructed appropriate demonstrations and instructions. Instruction was followed by three practice trials.

**Figure 2:**
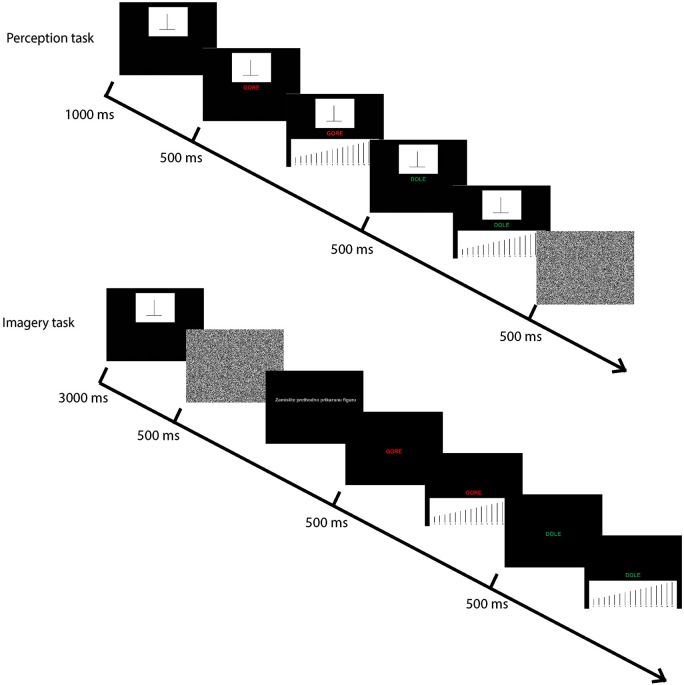
**The time sequence in the experimental trials**. Upper row shows the Perception task: both the stimuli and the measuring scale were presented on the screen during the estimation. Lower row shows the Imagery task: stimulus was removed after 3 s, and participants had to inspect their mental image and estimate the size on the presented scale.

In both tasks (perception and imagery), participants had to match the size of the two stimuli lines to the lines on the scale (Figure 2). That is, they had to choose one line from the scale that looked as if it was the same size as the target. However, in the perception task, the stimuli remained on the screen during the matching, while in the imagery task stimuli were removed.

Farrell (1979) noticed that participants had some difficulty distinguishing left and right targets on the screen. To test whether this was the case with our participants too, prior to Experiment 1, a pilot study was conducted with the aim of choosing the best labels for the target lines. The results revealed that our participants also had difficulties with labels but did not make mistakes when we used cue words in different colors. Therefore we used the labels “up” in red and “down” in green to cue them as to which part of the illusory figure they should match on a particular trial.

In the perception task, the stimulus was first shown on the upper part of the screen for 1000 ms, followed by the cue word indicating which line of the figure needed to be estimated (i.e., vertical or horizontal, labeled “up” and “down” respectively, but there was no fixation point). The scale was presented 500 ms later on the lower part and participants had to perform the matching task (without any time limit). After the matching task, the same procedure was repeated for the other line. At the end of each trial, a white noise mask was presented.

In the imagery task, stimuli were presented for 3000 ms, and then were removed. Participants had to visualize it,^3^ and only then they were cued which of the two lines will be estimated first (i.e., labels “up” and “down” would appear on the screen). During the matching task, only the labels and the scale were presented on the screen. The time for visualizing the figure was not limited.

During the whole trial, the participants were not asked to fixate at any particular part of the screen.

The illusion was presented ten times during the session, while pseudo illusion and distractor were shown two times. The order of presentation was randomized for each participant.

Apart from control stimuli we also used an attention test. Four times during the experiment, participants were asked to specify whether they were supposed to estimate the horizontal or vertical line first (i.e., “up” or “down”). That is, after a specific trial, a probe question would appear and the participants would have to report whether they were judging horizontal or vertical lines on that trial. This would help us establish if they were attending to each specific trial task.

Participants did the experiment individually, in the presence of an experimenter. Each experimental session lasted about 25 min.

## Results

A control analysis was performed (on perception task data) to assess responding strategies and possibly exclude participants that had the tendency to pretend to see illusory figures as composed of physically equal lines. Participants that did not use any responding strategy should exhibit the following patterns of results for: (1) pseudo-illusions, a significantly longer vertical line; (2) illusions, a significantly longer vertical line; and (3) distractors, no significant difference, or vertical line estimated as longe.

One of the 27 subjects was excluded because she failed to report the control stimuli correctly. The remaining 26 subjects passed all the controls and scored 81% on the attention test.

Their data were first analyzed using a two-way factorial ANOVA of Modality (perception/imagery) and Gender (male/female). The ANOVA was applied to the differences between their horizontal and vertical matches, following previous studies (Wallace, 1984a,b; Reisberg and Morris, 1985; Giusberti et al., 1998). The results (Table 2) showed that there was no significant difference in illusion size for the factors Modality, Gender, and their interaction. Critically, perception and imagery produced the same magnitude of illusion, 86%, since (on average) 38 mm horizontals were matched to 44 mm verticals.

**Table 2:** **ANOVA results for difference analysis in Experiment 1**.

Factors	df	df	F	*p*-level
Modality	1	257	0.589	0.443
Gender	1	257	2.252	0.135
Modality * Gender	1	257	0.245	0.620

A different insight into the nature of these phenomena can be obtained by using matches for each of the two lines separately (i.e., analysis of size). A line-size-match is a typical variable in psychophysical experiments, and it simply refers to the intensity estimation of perceived (or imagined) stimuli. Permitting Line orientation (horizontal, vertical) to be an additional factor, the magnitude of the illusion was still independent of modality, and there were no interactions, but gender now had an effect.

The three-factors ANOVA showed the following results (Table 3).

**Table 3:** **ANOVA results for size estimation in Experiment 1**.

Factors	df	df	F	*p*-level
Modality	1	257	2.88	0.090
Gender	1	257	21.15	0.000*
Line position	1	257	919.71	0.000*
Modality * gender	1	257	3.56	0.060
Line position * modality	1	257	0.59	0.443
Line position * gender	1	257	2.25	0.135
Line position * modality * gender	1	257	0.25	0.620

* p < 0.01.

**Figure 3:**
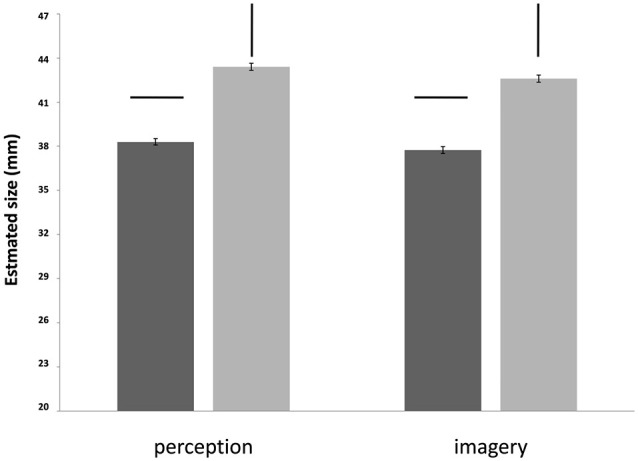
**The results for line estimations in the two tasks, perception task on the left and imagery task on the right**. Illusion strength is independent of Modality. Horizontal and vertical lines (above the histogram) denote the horizontal and vertical target lines in the stimuli.

Results showed no significant interactions between Line position, Gender and Modality (Table 3). Line position was statistically significant confirming the presence of the illusion (Figure 3). Most importantly, there was again no main effect of Modality showing no difference between the perception and imagery task in respect to line size. A significant main effect of Gender indicated that there are some gender differences in general processing, but this does not vary as a function of Modality or Line size (no interaction between the factors Line position and Gender, or Modality and Gender).

The stimuli size was 44 mm in all shown cases but judge correctly only by the female participants when positioned vertically (in both task modalities).

**Figure 4:**
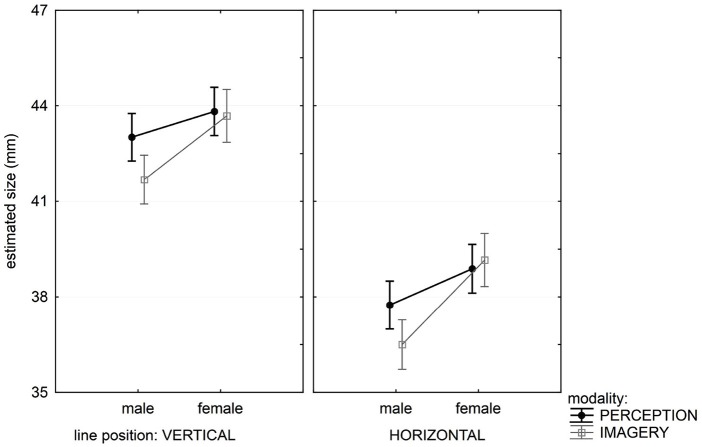
**Results in Experiment 1 (analysis of size)**. The estimation on the left graph depict the data for the vertical line which is estimated statistically significantly longer than the horizontal line, presented on the right portion of the graph. The two lines on each graph present the two task: perception and imagery. Vertical bars denote 0.95 confidence intervals.

The lack of an interaction between Line position and Modality indicated that the illusion intensity was independent of modality: that is, the illusion intensity was the same in perception and imagery (Figure 4). All results showed a stable effect of illusion, independent of gender or modality.

## Experiment 2

The factor Gender produced inconsistent results in the two analyses performed in Experiment 1. This factor was significant in the analysis that closely resembles a psychophysical matching task, where gender differences are usually not observed.

Experiment 2 was designed to further explore the issue of gender differences. We again used the horizontal-vertical illusion, but now tested three different stimulus sizes.

### Method

#### Participants

Eleven male and eleven female students of Psychology took part in the experiment. All of them had normal or corrected to normal vision and were naïve regarding the experimental hypotheses. They received course credit for their participation and signed consent form prior to the beginning of the experiment.

#### Stimuli

The horizontal-vertical illusion was constructed in three sizes: 31 mm, 51 mm and 67 mm (visual angle: 2°, 3.4°, 4.5°). Lines were 1 mm wide. Stimuli were placed on a white background (129 × 94 mm, visual angle: 10.4°× 7.6°). Each of the three illusions was presented ten times during the experiment.

Control stimuli (pseudo-illusions and distractors) were also constructed in three different sizes (Table 4). Each control stimuli was presented twice in the experiment.

**Table 4:** **Size of lines for pseudo-illusion and distractor stimuli (in millimeters)**.

	Small		Medium		Large	
	Vertical	Horizontal	Vertical	Horizontal	Vertical	Horizontal
**Stim. series**	**Pseudo-illusions**					
1	35	31	55	51	71	67
2	31	27	51	47	67	63
3	35	27	55	47	71	63
4	39	27	59	47	75	63
5	39	31	59	51	75	67
	**Distractors**					
1	31	35	51	55	63	71
2	27	39	55	51	67	71
3	31	39	51	59	63	75

#### Scale

To accommodate the change in stimuli size introduced in this experiment, a new scale with 16 vertical lines was used for matching. The shortest line was 19 mm, and the longest line was 79 mm, with each line being 4 mm longer than the previous.

#### Procedure

The procedure was identical to Experiment 1.

## Results

Control analysis did not reveal any response strategies and the attention task was successful (participant had 87% or more correct responses). Therefore the data from all of the participants entered the main analysis.

A four-way ANOVA with the following factors was used: Modality (perception/imagery), Gender (male/female), Stimuli size (small/medium/large) and Line position (horizontal/vertical). Results are summarized in Table 5.

**Table 5:** **Results of ANOVA for variable *estimation***.

Factors	df	df	F	*p-level*
Modality	1	202	1.71	0.193
Gender	1	202	1.93	0.166
Stimuli size	2	404	1384.43	0.000*
Line position	1	202	1353.44	0.000*
Modality * gender	1	202	45.46	0.000*
Stimuli size * modality	2	404	19.08	0.000*
Stimuli size * line position	2	404	30.52	0.000*
Stimuli size * gender	2	404	0.02	0.980
Line position * modality	1	202	1.28	0.259
Line position * gender	1	202	2.23	0.137
Line position * modality * gender	1	202	0.62	0.432
Stimuli size * modality * gender	2	404	8.80	0.0001*
Stimuli size * line position * modality	2	404	1.09	0.338
Stimuli size * line position * gender	2	404	0.80	0.451
Stimuli size * line position * modality *gender	2	404	0.50	0.606

* p < 0.01.

**Figure 5:**
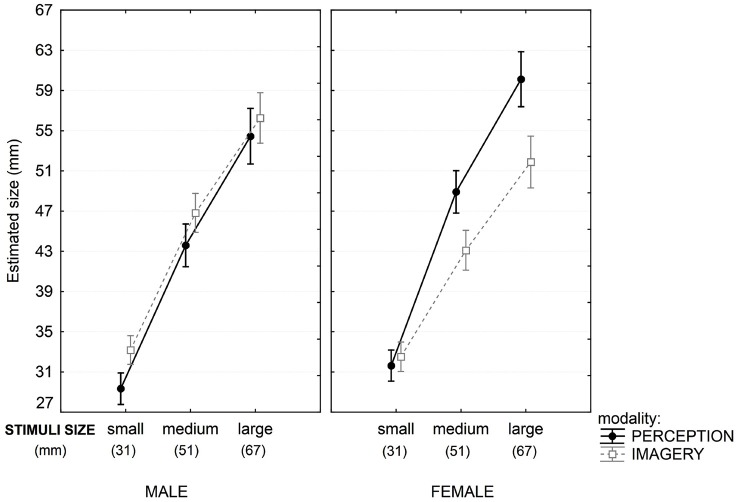
**Three-way interactions in the analysis of size in Experiment 2**. Results show clear difference between male (left) and female (right) participants in the two tasks (depicted by the two lines on the graph). The discrepancy between the two task increases on the right graph with stimuli size. Vertical bars denote 0.95 confidence intervals.

The most interesting result is a significant 3-way interaction between Stimuli size, Modality and Gender, indicating that depending on the size, male and female participants produced different matches in perception and imagery task (Figure 5).

Male participants estimated size equally in perception and imagery task, and Scheffe’s test showed that there was no significant difference in perception and imagery for any stimulus size. For female participants, there was no significant difference in the perception and imagery tasks for small stimuli, but differences appeared for medium (Scheffe test, *p* < 0.003) and large stimuli sets (Scheffe test, *p* < 0.000). Figure 5 shows that those differences increased with increasing stimulus size.

The non-significant 4-way interaction Stimuli size * Line position * Modality * Gender is also theoretically interesting. It provides an additional insight into the phenomena, revealing that the significant 3-way interaction (Stimuli size * Modality * Gender) is the same for both lines, horizontal and vertical. This probably means that the illusion strength is independent of other factors.

Since other significant 2-way interactions are already included in higher order 3- and 4-way interactions, they will not be separately described. However, it is important to the main effects. First the main effect of Line position, showed the existence of the basic illusion: vertical lines were estimated as longer. Stimuli size, confirmed the effect of physical size: lager stimuli were estimated as larger. The lack of a significant main effect of Modality confirmed our main hypothesis: in general, there is no significant difference in perception and imagery. Differences could only be observed in interaction with other factors (Gender and Stimuli size). The non-significant effect of Gender is in contradiction with the results we obtained in Experiment 1 but is in accordance with the psychophysical matching task.

## Discussion

In this study we focused on a comparison between perception and mental imagery. This relationship is at the heart of a long debate that either equates imagery to perception, postulating that mental images are (1) like percepts; (2) involve similar process; and (3) the same brain areas or that suggests that “the process of imagistic reasoning involves the same mechanisms and the same forms of representation as are involved in general reasoning” (Pylyshyn, 2002, p.158). The debate was aimed to resolve one of the major questions of cognitive psychology: the format of mental representations. Therefore, demonstrating that the same or similar processes are involved both in perception and imagery would strongly suggest the existence of same or similar representations.

Although there has been relatively little behavioral research comparing directly perception and imagery though for an overview of earlier work, see the seminal paper by Finke and Shepard (1986), numerous results from neuroscience support the view that perception and imagery are based on similar processes. Our research hypotheses were inspired by those results. This study aimed to explore the similarity of perception and imagery, but using behavioral methodology, i.e., processing of visual illusions.

We considered several flaws in the reported literature and tried to resolve most of the methodological issues. A simple experiment with clearly separated imagery and perception tasks was conducted, applying the same size-measurement technique in both cases. Simple, non-ambiguous illusory figures were used, answering strategies were controlled, the influence of afterimages was prevented, and data for both high- and low-imagers were collected. A possible influence of the experimenter’s expectation or suggestion was almost completely reduced.

Introducing two control sets of stimuli provided an important advantage in our experiment. It allowed us to identify reasons for failure to experience an illusion, and to exclude participants that used answering strategies, both in perception and imagery. By controlling all conditions in the experiment, we avoided possible misinterpretation of imagery results, allowing us to conclude that illusions can be experienced in imagery.

In the first experiment we demonstrated that the horizontal-vertical illusion could be created in imagery. A specific type of variable, the difference between the lines, enabled us to determine the precise size of the illusory effect in imagery and to compare it with typical perceptual results. Our findings revealed that there was no significant difference in perception and imagery—the difference between the horizontal and vertical line was the same for perception and imagery. Thus, our results support some of the previously reported results, for example Wallace’s findings (Wallace, 1984a,b).

In this study we also obtained the first quantitative measure of illusion size in imagery. This measure was independent of participants’ judgments on the equality of lines, so the results were not influenced by instruction and experimenter expectation, which was one of the main flaws in previous studies (Wallace, 1984a,b; Reisberg and Morris, 1985). This kind of measurement enabled us to compare size in perception and imagery directly.

There might be another important source of difference in measured size between the two tasks that is not a consequence of imagery but the underlying memory processes. Imagery tasks by definition involve memory and memory representations might be noisier or lower resolution than those created in perception (Bartlett, 1932; but see also Potter, 1976). Furthermore, details and elements in memory are always likely to be subject to a certain degree of decay (Ebbinghaus, 1964). In the Introduction we also mentioned other more sophisticated memory effects. One such effect is boundary extension that leads an observer to “remember seeing more of a scene than was shown” (Intraub et al., 1996). This research specifies that the effect only involves scene perception, creating activation in para-hippocampal place area (PPA) and retrosplenial cortex (RSC), but never in the areas involved in object perception, i.e., lateral occipital complex (LOC; Park et al., 2007). This implies that boundary extension affects view-boundaries and not object-boundaries (Gottesman and Intraub, 2003). Furthermore, Gottesman and Intraub (2002, 2003) offer an explanation of boundary extension that relies on amodal perception and in that respect is unlikely to apply to our, fully presented, stimuli. Finke et al. have found similar memory distortions in the dynamic domain (e.g., Freyd and Finke, 1984). As all of our stimuli were static, and did not imply motion, we would not expect these dynamic effects to occur. Finally, our results are very systematic in both the perceptual and imagery domains, never revealing any of the differences suggestive of memory representation biases. Our Figures 3 and 5, for example, directly compare magnitude estimation for the same line presented in perception and retrieval from memory in the imagery task. This is further confirmed by the non-significant statistical tests in a variety of conditions (factor Modality, Tables 2, 3, 5). As we essentially found no differences between performance measured in our two tasks, it seems unlikely that memory processes are significantly modulating the current results.

While we were analyzing size and the distribution of results, we noticed that female participants performed somewhat differently in imagery. This was a tendency, not a significant result. Although this is not a typical finding for cognitive tasks we did obtain similar results in one previous imagery study (Stojanović and Zdravković, 2007). Thus we designed a second experiment with the aim of further exploring gender differences in size, in both perception and imagery. The measuring scale and stimuli sets were adapted in order to provide more precision.

The second experiment confirmed the main results of the first experiment: there was no significant difference between perception and imagery in illusion intensity. Also, there were no gender differences in illusion intensity. But the second experiment gave us further insight into the nature of the phenomena of mental imagery. Although the illusion is the same strength in both modalities, it appears that the size of the images in perception and mental images are not the same for both genders. Male participants perform equally in perception and imagery for all stimuli sizes. But female’s estimation depended on stimulus size: there was no significant difference in perception and imagery for small stimulus size (which were comparable to those used in Experiment 1), but there were increasing differences for medium and large stimuli. In other words, female tend to underestimate the size in imagery and this tendency increased with increasing stimulus size.

Our results concerning the relationship between perception and imagery are fully in accordance with those of Kosslyn et al. (1997, 1999, 2001); Ganis et al. (2004). In his theory, Kosslyn (2005) proposed that mental imagery and perception are based on similar underlying cognitive components, and postulates six major systems involved in perception and imagery: visual buffer, attention window, processing object properties vs. spatial properties, associative memory, information shunting and attention shifting system. A possible explanation for our results might relate to properties of the associative memory system. Kosslyn argued that both propositional and analog representations are placed in associative memory, while images in the visual buffer are pictorial in nature. That implies that both types of information, pictorial and verbal, are used in the process of imagery. Thus, we can suppose that difference between male and female in imagery could be a consequence of different ratios of pictorial and verbal elements for specific object in associative memory. Since the mental image of male participants is “equal” to the image in perception, it can be assumed that they use more pictorial information during imagery. Conversely, female participants may rely more on verbal descriptions. Therefore, female participants’ tendency to reduce image size in imagery could be a result of an energy saving strategy: since verbal descriptions are used, forming a smaller image uses less energy and memory capacity. However, those explanations are purely hypothetical and demand further empirical exploration.

Cognitive abilities research supports the view of gender differences in spatial ability. Meta-analysis of gender differences in spatial abilities has shown that male participants perform better in mental rotation, spatial perception and spatial visualization (Voyer et al., 1995). Some later results suggest that male outperform female participants on spatial-ability tests, although differences within groups are larger than between groups (Weiss et al., 2003a). Moreover, Weiss et al. fMRI study showed different brain activation during their mental rotation task: male participants showed stronger parietal activation, while female participants showed greater right frontal activation (Weiss et al., 2003b). Our own previous research is inconsistent: sometimes we found differences (Stojanović and Zdravković, 2007) and sometimes we did not (Ćirović and Zdravković, 2011).

Based on those findings, we can suppose that male and female participants perform differently on spatial tasks, including imagery, but further research in this area should include more specified and detailed imagery tasks.

## Conclusion

Analyzing participants’ size estimations enabled us to measure illusion in imagery. Moreover, it enabled us to explore some of the main characteristics of mental images. This approach helped us to test our initial hypothesis, so we demonstrated that illusion could occur in imagery. In addition, we found that illusion intensity is the same in perception and imagery. Although we found no significant difference in illusion intensity, gender differences appeared in mental images: female participants tend to make images of the smaller spatial format compared to images in perception. However, the origin of those differences cannot be explained within our study, but require further exploration.

## Conflict of interest statement

The authors declare that the research was conducted in the absence of any commercial or financial relationships that could be construed as a potential conflict of interest.

## Footnotes

^1^Original instruction (Wallace, 1984a; page 157): “…the subjects were told to imagine a line of the same physical length as the horizontal one present and to imagine their line as being located below the one that they could see… Can you now tell me if the top line appears to be longer than the bottom line, if the bottom line appears to be longer than the top line, or if the two lines appear to be equal in length?”

^2^This does not include the RT and accuracy studies, but reffers only to the spatial characteristics of the the imagined illusions.

^3^The instruction was given in Serbian and the crucial part (“vaš zadatakće biti da zamislite figuru koja vam je prethodno bila prikazana na ekranu”) could be literally translated as “your task will be to imagine the figure that has just been presented to you on the computer screen”.
